# Septic cutaneous emboli revealing a severe case of *Klebsiella pneumoniae* liver abscess syndrome

**DOI:** 10.1099/jmmcr.0.005148

**Published:** 2018-04-12

**Authors:** Florentina Paraschiv, Gabriel Adrian Popescu, Alina Maria Borcan

**Affiliations:** ^1^​ National Institute for Infectious Diseases Matei Bals, Bucharest, Romania; ^2^​ Infectious Diseases, Carol Davila University of Medicine, Bucharest, Romania

**Keywords:** *Klebsiella pneumoniae* liver abscess syndrome, cutaneous emboli, meningitis, severe thrombocytopenia, antibiotic and abscess drainage

## Abstract

**Introduction:**

*Klebsiella pneumoniae* liver abscess syndrome (KLAS) is characterized by bacteraemia, liver abscesses and metastatic infection caused by a hypervirulent strain of *Klebsiella pneumoniae*, usually belonging to the capsular serotype K1 or K2. Initially, KLAS was described in Eastern Asia; recently isolated cases have been reported from different parts of the world.

**Case presentation:**

We describe the case of a woman with KLAS including meningeal, ocular and cutaneous metastatic infection and organ dysfunctions (coagulation abnormalities, thrombocytopenia and increased creatinine level). The identification of a hypermucoviscous strain of *Klebsiella pneumoniae* was possible by culture from one of the cutaneous emboli and subsequently confirmed by blood cultures. The patient fully recovered after abscess drainage and prolonged antibiotic treatment.

**Conclusion:**

We have pointed out about the importance of sampling each septic focus in order to identify the aetiology of a disseminate infection.

## Introduction

*Klebsiella pneumoniae* liver abscess syndrome (KLAS) is a clinical syndrome characterized by bacteraemia, liver abscesses and metastatic infection caused by a hypervirulent strain of *Klebsiella pneumoniae*, usually belonging to the capsular serotype K1 or K2 [1]. Described mostly in Taiwan, KLAS has been frequently found and documented in Eastern Asia, but in the last 15 years sparse cases have been reported from other regions, such as Europe and America, including Balkan countries [2, 3]. Extrahepatic metastatic infection at distant sites has been reported in 8.7–15.5 % of KLA patients, more often being metastatic meningitis or endophtalmitis, and rarely abscesses in the lung, prostate, skin or bone [1, 4]. Previous studies have identified diabetes mellitus as a risk factor for KLAS [5]. The first case of *Klebsiella pneumoniae* liver abscess described in Romania was in 2014 at the National Institute of Infectious Diseases ‘Professor Dr Matei Bals’ Bucharest [6].

## Case report

**Fig. 1.**
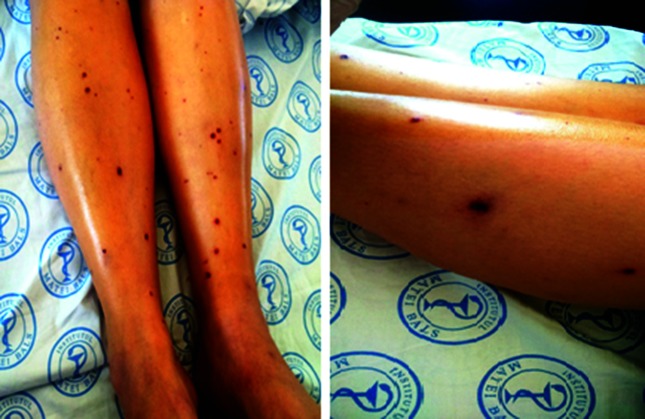
Lower limbs with septic cutaneous emboli.

A 53-year-old woman was admitted to the infectious diseases department presenting with a 24 h history of high fever, chills, vomiting, headache, confusion and altered general condition. The patient had a cholecystectomy several years ago and in the last month was evaluated for a possible diabetes mellitus. The patient had no history of travelling in foreign countries in the past five years. At admission the clinical examination revealed a partially conscious, delirious patient with nuchal rigidity, oliguria and haematuria. The skin was pale, with several bilateral ecchymoses on the legs and arms and papular exanthema on the calf (Fig. 1). The ophthalmic examination showed retinal haemorrhage and uveitis in the right eye.

Laboratory test showed leukocytosis (19 700 cells mm^−3^) with neutrophilia (18 700 cells mm^−3^) severe thrombocytopenia (7000 cells mm^−3^), high d-dimers (15 505 ng ml^−1^), inflammation (fibrinogen 8.1 g l^−1^, serum procalcitonin 60.3 ng ml^−1^), creatinine (406.7 µmol l^−1^), urea (244.5 mg dl^−1^), hyponatraemia (131 mmol Na l^−1^), hypokalaemia (3.3 mmol K l^−1^), cytolysis: aspartate aminotransferase 1.1× upper limit of normal (ULN), alanine aminotransferase 1.5× ULN, increased gamma-glutamyl transferase level (3× ULN), hypoalbuminaemia (2.5 g dl^−1^) and hyperglycaemia 14.2 (mmol l^−1^).

Because of the haemorrhagic risk, the lumbar puncture was delayed, but we microbiologically sampled the skin lesions on her calves and we took three sets of blood cultures. Treatment with vancomycin and ceftriaxone was started. We used insulin in order to normalize hyperglycemia and collyrium with dexamethasone, antibiotics and an anticholinergic for eye involvement.

A head magnetic resonance imaging (MRI) examination was performed, showing diffuse oedema and a minor lenticular lesion, possible an infarction. The echocardiography did not retrieve endocardial lesions. Abdominal echography revealed a liver mass and MRI was performed that showed an abscess with dimensions of 9.0×6.0 cm in the VIIth liver segment. Considering the probable involvement of a Gram-negative rod, the antibiotic treatment was changed to meropenem (2 g every 8 h).

On the second day of hospitalization the cultures performed from the skin lesions yielded Gram-negative lactose-fermenting rods, subsequently identified as *Klebsiella pneumoniae* through MALDI–TOF MS. The isolated strain had a positive string test, indicating a possible hypermucoviscosity phenotype. The antimicrobial susceptibility test yielded an isolate susceptible to cephalosporins, aminoglycosides and fluoroquinolones and we changed the antibiotic therapy to ceftriaxone (2 g every 12 h). On the third day of hospitalisation the blood cultures were also positive for *Klebsiella pneumoniae* with similar antimicrobial susceptibility.

On day 7, platelets count were 84 000 mm^−3^ and a lumbar puncture was performed, sampling a xanthochromic cerebrospinal fluid (CSF), with pleiocytosis, 15 leukocytes mm^−3^, elevated lactic acid (5.4 mmol l^−1^) and elevated proteins (2.54 g l^−1^). After 15 days of ceftriaxone (4 g per day) the patient underwent percutaneous drainage of the hepatic abscess. Combined therapy, both antibiotic and surgery, leaded to important improvement of the patient. The antibiotherapy was switched to oral amoxicillin–clavulanate (1 g three times a day) and the patient was discharged with close follow up until total remission of the hepatic abscess was confirmed through abdominal echography. Three months later she had good glycaemic control with oral medication and almost normal visual acuity.

## Discussion

The literature records that in the last 20 years *Klebsiella pneumoniae* has become the most common pathogen causing pyogenic liver abscesses in Singapore and Taiwan [7]. The *Klebsiella pneumoniae* isolates from KLAS patients belong to the capsular serotype K1 or K2, two hypervirulent types [8]

Recently, KLAS cases have been reported in Europe and Americas in patients without a history of travelling in Eastern Asia; hence, patients outside the initial reporting area are not protected [9]. In Romania this is the second published case of KLAS and the first one with this kind of metastatic determinations. The hypermucoviscous character of the isolated strain was confirmed through microbiological string testing. It is important to mention that the aetiological diagnosis was established through culturing from the skin septic metastasis and supported by the blood cultures. Microbiological sampling of any possible septic foci is the most important step for the diagnosis of severe infections and for subsequent targeted antibiotic therapy. Sampling of septic foci needs to be performed before starting antibiotic treatment to increase the chance of identifying and characterizing the pathogen.

Usually, in the liver abscesses aetiology, polymicrobial involvement is common, but *Escherichia coli* and *Klebsiella pneumoniae* are the two most frequently isolated pathogens [10]. In our case, KLAS was suspected before microbiological diagnosis on the basis of clinical features, the presence of multiple septic foci in the absence of currently existing bowel disorders. Another important clue was the medical data regarding the diabetes mellitus. It is a well documented fact that KLAS is frequently associated with disorders of carbohydrate metabolism, in particular clinical forms with endophtalmitis [5].

KLAS is an aggressive, complex pathology with a great possibility of generating a wide range of complications and sequelae. It is however important to note that the hypervirulent *Klebsiella pneumoniae* serotypes retained sensitivity to antibiotics [11, 12]. The strain isolated from our patient fitted the mentioned characters.

Considering the high risk of pairing the hypervirulence with antimicrobial resistance of pathogens, it is very important to maintain as long as possible the ‘wild-type’ antimicrobial susceptibility for these K1 and K2 strains. Recently, a few case reports have described K1 isolates with multi-drug resistance to antibiotics [13, 14]. The antimicrobial characterization helps with prescribing targeted antimicrobial medication and sparing wider-spectrum and sometimes last-line antibiotics; it is the best approach to diminish the risk of transforming these strains into more drug-resistant germs.
